# Supplementary material for: Asynchronous magnetic resonance elastography: Shear wave speed reconstruction using noise correlation of incoherent waves
Source: Magn Reson Med. 2022 Oct 27;89(3):990–1001. doi: 10.1002/mrm.29502 (PMC9792433; doi:10.1002/mrm.29502)
Supplement: Supplementary file 1 — DATA S1 MATLAB code used to generate the numerical simulations of Figure 1 [file MRM-89-990-s002.zip › k-Wave/helpfiles/getFDMatrix.html]

getFDMatrix :: Functions (k-Wave)


# getFDMatrix

Create a matrix of finite-difference coefficients.

## Syntax

```
fdm = getFDMatrix(Nx)
fdm = getFDMatrix(Nx, dx)
fdm = getFDMatrix(Nx, dx, deriv_order, accuracy_order)
```

## Description

`getFDMatrix` returns a matrix of finite-difference coefficients to compute the derivative of the given order with the given numerical accuracy. The coefficients in the center of the grid use centered differences, while the coefficients at the edges of the grid use forward and backward differences. The matrix is generated and returned in sparse format. To convert to a full matrix, use the syntax fdm = full(fdm).

Example: Compute the gradient of a 1D column vector

```
dx = pi/20;
x = (0:dx:4*pi).';
y = sin(x);
fdm = getFDMatrix(length(x), dx);
dydx = fdm * y;
plot(x, y, 'k-', x, dydx, 'r-');
```

## Inputs

|  |  |
| --- | --- |
| `Nx` | size of data to differentiate |

## Optional Inputs

|  |  |
| --- | --- |
| `dx` | spacing between grid points (default = 1) |
| `deriv_order` | order of the derivative to compute, e.g., use 1 to compute df/dx, 2 to compute df^2/dx^2, etc. (default = 1) |
| `accuracy_order` | order of accuracy for the finite difference coefficients. Because centered differences are used, this must be set to an integer multiple of 2 (default = 2) |

## Outputs

|  |  |
| --- | --- |
| `fdm` | matrix of finite difference coefficients |

## See Also

`gradient`, `gradientFD`, `gradientSpect`
